# A Single Multiplex CytoBas Assay Incorporating Eight Major Components for Accurate Detection of Allergen Sensitization in Asthma and Allergic Rhinitis

**DOI:** 10.1111/all.16513

**Published:** 2025-03-07

**Authors:** Lin Hsin, Mark Hew, Pei Mun Aui, Kirsten Deckert, P. Mark Hogarth, Robyn E. O'Hehir, Menno C. van Zelm

**Affiliations:** ^1^ Department of Immunology, School of Translational Medicine Monash University Melbourne Victoria Australia; ^2^ Allergy, Asthma and Clinical Immunology Alfred Health Melbourne Victoria Australia; ^3^ Public Health & Preventive Medicine Monash University Melbourne Victoria Australia; ^4^ Immune Therapies Group Burnet Institute Melbourne Victoria Australia; ^5^ Department of Clinical Pathology The University of Melbourne Parkville Victoria Australia; ^6^ Department of Immunology, Erasmus MC University Medical Center Rotterdam the Netherlands

**Keywords:** allergic rhinitis (AR), atopic asthma, component‐resolved diagnosis, grass pollen, house dust mite, pet allergy

## Abstract

**Background:**

Allergic rhinitis and asthma can be triggered by a variety of aeroallergens, including house dust mites (HDM), tree and grass pollen, and household pets. Identification of the relevant allergen is critical for lifestyle changes and treatments, including allergen immunotherapy. We here assessed the diagnostic performance and clinical utility of a single flow cytometry staining of basophils with major aeroallergen components (AeroDiff CytoBas).

**Methods:**

In 156 atopic patients with allergic rhinitis/asthma and 21 non‐atopic individuals, allergen‐specific IgE levels were determined by ImmunoCAP, and component‐specific IgE by ELISA. PBMCs were analyzed by flow cytometry with basophil markers and eight fluorochrome‐conjugated allergen component tetramers.

**Results:**

Patients were stratified for sensitization to each of the four allergens. Allergen‐component staining in a single multiplex CytoBas assay and component‐specific IgE serology performed similarly for Der p 2, Lol p 1, Fel d 1, and Can f 1 (ROC AUC: 0.76–0.97 vs. 0.73–0.93). CytoBas had greater diagnostic accuracy than component‐specific IgE serology (*p* < 0.001) for HDM sensitization using Der f 1 or Der p 1, and grass pollen using Lol p 5 or Phl p 1. Furthermore, the combined evaluation of Der p 1 and Der p 2 with CytoBas was 96.3% sensitive and 90.7% specific for HDM sensitization. The combined evaluation of Lol p 1 and Lol p 5 achieved 95.4% sensitivity and 96.4% specificity for ryegrass pollen sensitization.

**Conclusion:**

AeroDiff CytoBas has similar to superior diagnostic accuracy compared to singleplex IgE serology, with the additional advantage of a single assay to evaluate multiple allergens. This enables precise and efficient component‐resolved diagnosis of aeroallergen sensitization to guide personalized treatment for patients with allergic rhinitis and/or asthma.

## Graphical abstract

- Combined evaluation of 8 aeroallergen components in a single flow cytometry assay can differentially detect house dust mite, grass pollen, cat, and dog sensitization on blood basophils (AeroDiff CytoBas).
- The AeroDiff CytoBas assay demonstrated similar to superior diagnostic accuracy compared to singleplex component‐specific IgE serology.
- Using two components per allergen, AeroDiff CytoBas accurately detected house dust mite (96% sensitivity and 91% specificity) and grass pollen sensitization (95% sensitivity and 96% specificity).

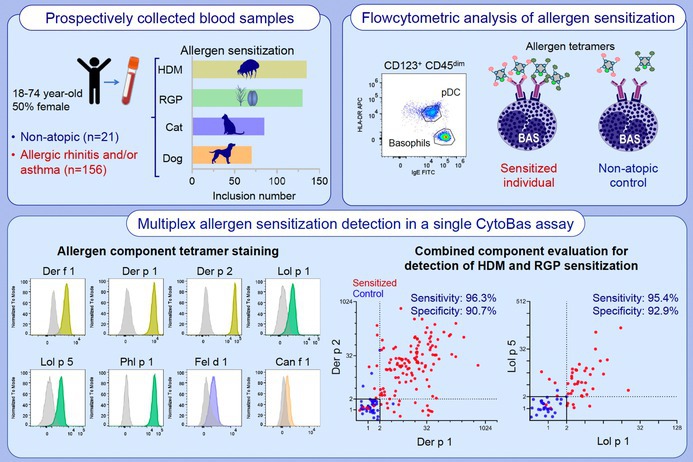

**Abbreviations**
AIT: allergen immunotherapy
APC: allophycocyanin
AR: allergic rhinitis
AUC: area under the curve
BAT: basophil activation test
CRD: component‐resolved diagnostics
HDM: house dust mite
MFI: median fluorescence intensity
MOI: multiplicity of infection
RGP: ryegrass pollen
ROC: receiver operating characteristic
SPT: skin prick test

## 1 Introduction

Atopic asthma affects around 262 million people worldwide, including 2.8 million Australians (11% of the population) [1, 2]. Allergic rhinitis (AR) often accompanies asthma and, if not well managed, may exacerbate asthma [3]. Both are prevalent chronic respiratory conditions that significantly impact the quality of life and healthcare costs globally [4]. Patients display a type I IgE‐mediated hypersensitivity to environmental particles (aeroallergens) and are frequently polysensitized to multiple aeroallergens [5]. Allergen immunotherapy (AIT) is the only disease‐modifying treatment, and it induces immunological changes with sustained benefits for asthma and AR patients [6]. However, considerable barriers remain to effective AIT utilization, which involves several years of treatment and considerable cost [7]. For patients suffering from these respiratory diseases, accurate diagnosis of allergen sensitization is crucial for effective management and treatment, including AIT [8, 9].

Current first‐line diagnostics for the detection of allergen sensitization in patients with AR and/or asthma include skin prick testing (SPT) and/or measurement of serum‐specific IgE [10]. These tests typically use unfractionated allergen extracts, resulting in limitations in specificity [11] and inconsistency in the ultimate diagnosis [12]. The tests cannot distinguish primary sensitization from clinical cross‐reactivity to specific allergens, which may confound diagnosis and treatment [13, 14]. Employing allergen components can overcome these limitations, and component‐resolved diagnostics (CRD) are increasingly used [15], with up to 300 allergen components evaluated in a serum IgE panel test [16, 17]. The molecular sensitization profiles generated by such multiplex CRD can guide clinicians toward more effective treatment decisions [18, 19].

Serum IgE‐based CRD can offer improved sensitivity and specificity but may not always reflect the functional relevance of specific IgE [20], as it is cell‐bound IgE on mast cells and basophils that mediates the allergic response. The basophil activation test (BAT) is an in vitro functional assay that can be utilized to detect the presence of allergen‐specific IgE on immune effector cells and the capacity to induce cellular degranulation [21, 22]. However, the BAT requires processing of blood within a few hours (up to 24 h) for optimal basophil responses [23]. This can become labor‐intensive, which has hindered its routine use and large‐scale implementation [23]. We recently developed a flow cytometric assay that enables detection of allergen sensitization on blood basophils using fluorescently labeled recombinant allergen component tetramers (CytoBas) [13, 24]. These allergen tetramers can activate basophils upon in vitro exposure to allergen‐specific IgE, enabling CytoBas to detect functional allergen sensitization [13, 24]. The CytoBas approach showed greater than 90% sensitivity and specificity for the detection of allergen sensitization to two major triggers of asthma and AR: HDM and RGP, respectively [13, 24].

The most prevalent triggers for AR and atopic asthma in the State of Victoria in Australia are HDM, ryegrass pollen (RGP), cats, and dogs [25, 26, 27]. The dominant HDM species in Australia is *Dermatophagoides pteronyssinus*, and its major allergen components are Der p 1 and Der p 2 [28]. Lol p 1 and Lol p 5 are the major RGP components, and 90% of RGP allergic patients in Australia are sensitized to one or both components [13, 29]. Cat and dog allergies are increasingly prevalent globally (10%–20%) and in Australia [30, 31]. The major components, Fel d 1 (cat) and Can f 1 (dog), are significant positive predictors of the respective allergies [32, 33].

We aimed to determine the diagnostic capability of a multiplex AeroDiff CytoBas assay in a Victorian patient cohort using the major components from the four main triggers of aeroallergy: HDM (Der p 1 and Der p 2), ryegrass pollen (Lol p 1 and Lol p 5), cat (Fel d 1) and dog (Can f 1). To evaluate cross‐reactivity to homologous species and demonstrate global relevance, Der f 1 and Phl p 1 were included. *D. farinae* is the more prevalent HDM species in Asia and the US [34], and Der f 1 is 90% homologous to Der p 1 [35]. Phl p 1 is > 85% homologous to Lol p 1 [36] and is the major allergen component from Timothy grass, a prevalent species especially in Europe [37]. Using ImmunoCAP as the gold standard reference [38], the diagnostic performance of CytoBas was compared with allergen component‐specific IgE serology.

## 2 Methods

### 2.1 Study Participants and Sample Collection

Participants diagnosed with perennial/seasonal AR with or without asthma and serum‐specific IgE levels of ≥ 0.35 kUA/L to HDM, RGP, cat, and/or dog (ImmunoCAP, Phadia, Uppsala, Sweden) were recruited from the Allergy Clinic at the Alfred Hospital in Melbourne, Victoria, Australia. Non‐atopic donors were recruited based on the absence of clinical allergy and the absence of sensitization to HDM, RGP, cat, and dog (all < 0.35 kU_A_/L by ImmunoCAP). Exclusion criteria for all donors were: the presence of immunodeficiency or gastrointestinal complications, receipt of AIT within 5 years, and the use of prescribed beta‐blockers or oral corticosteroids. The use of symptom relievers for AR, such as antihistamines and topical corticosteroids, was permitted. Lithium‐heparinized blood samples were collected from all subjects and processed within 24 h for serum and PBMC isolation and cryopreservation for later batch analysis. The study was conducted in compliance with the Declaration of Helsinki, and written informed consent was collected from each participant prior to inclusion (Alfred Ethics, Project 297‐20).

### 2.2 Recombinant Protein Production and Protein Tetramerization

Production of recombinant Der f 1, Der p 1, Der p 2, Lol p 1, and Lol p 5 was described previously [13, 24]. In a similar fashion, protein sequences Phl p 1.0102, Fel d 1.0101 (chain 1 and 2) and Can f 1.0101 were obtained from the Allergen Nomenclature website (allergen.org) [39]. All constructs were generated with leader sequences for secretion, an Avi‐tag for biotin‐protein ligase (BirA)‐catalyzed biotinylation, and a 6‐His tag for cobalt affinity column purification (Figure S1A). Phl p 1 was designed with the same H104V mutation as Lol p 1 to remove its enzymatic activity (Figure S1A) [13]. Codon‐optimized DNA constructs for the arachnid and plant allergen components were cloned into the pFastBAC vector (Thermo Fisher Scientific, Waltham, Mass) before being incorporated into a Bacmid for baculovirus production. Bacmids were transfected into ExpiSF9 cells, and supernatant was collected after 4 days. The viral titers were determined with a flow cytometric baculovirus titering assay [40], and a new culture of ExpiSF9 was infected with a multiplicity of infection (MOI) at 0.3–0.5 and cultured at 27°C for protein expression.

The DNA construct encoding Fel d 1 was generated with the native leader sequence of chain 2 followed by the fused coding regions of chain 2 and chain 1 [41], and C‐terminal AviTag and 6‐His tag [42]. Can f 1 was generated with the N‐terminal human Ig leader sequence and the same C‐terminal AviTag and 6‐His tag [42]. These mammalian proteins were produced in the human embryonic kidney Expi 293 T cell line, as described previously [42].

All proteins were purified from culture supernatants on Cobalt columns, as described previously [24]. Purified proteins were then concentrated and dialyzed in NaCl (150 mM)‐containing TRIS (10 mM) buffer (TBS150) to enhance protein solubility (salting‐in) [43]. After dialysis, proteins were run under denaturing and non‐denaturing conditions on a 4% to 15% mini‐PROTEAN TGX Stain‐Free Protein Gel (Bio‐Rad Laboratories, Hercules, Calif), and following blotting, evaluated with a 6x‐His Tag mouse mAb (clone: HIS.H8; Thermo Fisher Scientific). The validated recombinant proteins were subjected to targeted biotinylation incubation with a BirA enzyme (*Escherichia coli* 6.3.4.15; Avidity LLC, Aurora, CO, USA) in SuperMix buffer (10×; Avidity LLC) at 4°C overnight [44], followed by dialysis against 10 mM TRIS (pH 8) to remove any unreacted biotin. The biotinylated proteins were tetramerized by adding fluorochrome‐conjugated streptavidin, at an allergen‐streptavidin molar ratio of 4:1, making unique aeroallergen tetramers: [Can f 1]_4_‐BUV395, [Lol p 5]_4_‐BUV496, [Der f 1]_4_‐BUV615, [Fel d 1]_4_‐BUV737, [Der p 2]_4_‐BV480, [Der p 1]_4_‐BV650, [Phl p 1]4‐BV711, and [Lol p 1]_4_‐PE.

### 2.3 Quantification of Allergen‐Specific Serum IgE Levels

Serum‐specific IgE to single allergen components was measured by in‐house ELISA [13, 42]. High‐binding EIA/RIA Assay Microplates (Sigma Aldrich, Burlington, MA, USA) were coated with recombinant Der f 1, Der p 1, Der p 2, Lol p 1, Lol p 5, Phl p 1, Fel d 1, or Can f 1 overnight at 4°C. Separate wells were coated with serial dilutions of recombinant human IgE‐kappa (clone AbD18705; Bio‐Rad, Puchheim, Germany) to generate the standard curve for interpretation of the serum IgE from samples. Plates were blocked with 5% skim milk powder in PBS and then incubated in diluted serum samples (1:20) from donors. The plate was then washed, and bound IgE was detected using polyclonal rabbit anti‐hIgE (Agilent, Santa Clara, CA, USA), which was then detected by a secondary polyclonal goat anti‐rabbit HRP (Promega, Madison, WI, USA). For Der p 1‐specific IgE measurements, a polyclonal human IgE cross‐adsorbed secondary antibody, HRP (Thermo Fisher) was used for the detection of IgE. ELISA plates were developed with TMB solution (Life Technologies, Carlsbad, CA), and the reaction was stopped with HCl (1 N; Chem‐Supply). The absorbance at OD450 nm from each well was measured with the Multiskan Microplate Spectrophotometer (Thermo Fisher).

### 2.4 Basophil Staining and Spectral Flow Cytometry

Individual vials of 1 mL cryopreserved PBMC (10 million) were thawed in a 37°C water bath until crystals arose, followed by the addition of 1 mL FCS and 8 mL of RPMI‐1640 (Sigma‐Aldrich). Samples were washed again with FACS buffer (0.7% BSA and 0.1% sodium azide in PBS) and then strained through a 70‐μm filter for cell count and viability determined with the automated cell counter (Nexcelom, Lawrence, MA, USA). 6–9 million cells of PBMC were incubated with the eight aeroallergen tetramers (1 μg each per 100 μL stain) in the dark for 5 min at room temperature. In a separate tube, the remaining 2 million PBMC were incubated with equal amounts of streptavidin‐only conjugates (BUV395, BUV496, BUV615, BUV737, BV480, BV650, BV711, and PE) [42, 45]. Both sample tubes were then incubated with the backbone antibodies CD45‐ PerCP‐Cy5.5 (2D1; BD Biosciences), CD123‐cFluorBYG781 (SK3; Cytek BioSciences, Fremont, CA), IgE–fluorescein isothiocyanate (polyclonal; Thermo Fisher Scientific), HLA‐DR–allophycocyanin (APC) (L243; BioLegend, San Diego, CA) and viability dye viaDye Red (Cytek BioSciences) (Table S1) in the dark for 15 min at room temperature. Subsequently, cells were washed with FACS buffer and fixed with 2% paraformaldehyde for 20 min at room temperature. After staining and fixing, cells were washed twice with 2 mL FACS buffer, then resuspended in FACS buffer to run on a five‐laser Cytek Aurora (Cytek Biosciences). The standard Cytek Assay Settings were employed and were adjusted daily utilizing the SpectroFlo Quality Control beads, according to the manufacturer's recommendations [46]. Light scatter settings were fine‐tuned for basophil identification, and the FSC area scaling factor was 1.23. Samples were acquired using the live unmixing function on SpectroFlo and were run at a flow rate of 3500–5500 events per second. PBMC viability ranged from 75%–95%, and this was > 95% for basophils.

### 2.5 Data Analysis and Statistics

Statistical analyses of flow cytometric data were conducted with GraphPad Prism (v9.0.1; GraphPad Software, Dotmatics, Boston, MA, USA). The nonparametric Wilcoxon signed‐rank test was used for paired data, and the nonparametric Mann–Whitney *U*‐test was used for non‐paired data. Receiver operating characteristic (ROC) curves were created to determine the performance capabilities (specificity and sensitivity) of allergen components in serology and CytoBas assays between sensitized patients and controls (non‐sensitized and non‐atopic). The nonparametric Delong test was used to compare the area under the curve (AUC) values obtained from ROC analysis between the CytoBas and serology tests. Correlation analyses between CytoBas and serology assays were conducted using Spearman's rank correlation test to assess monotonic relationships. Subsequent post hoc analyses involved log–log non‐linear fitting, and correlation was determined by the coefficient of determination (*r* ^2^). For all tests, *p* < 0.05 was considered statistically significant.

## 3 Results

### 3.1 Study Participant Characteristics

**FIGURE 1:**
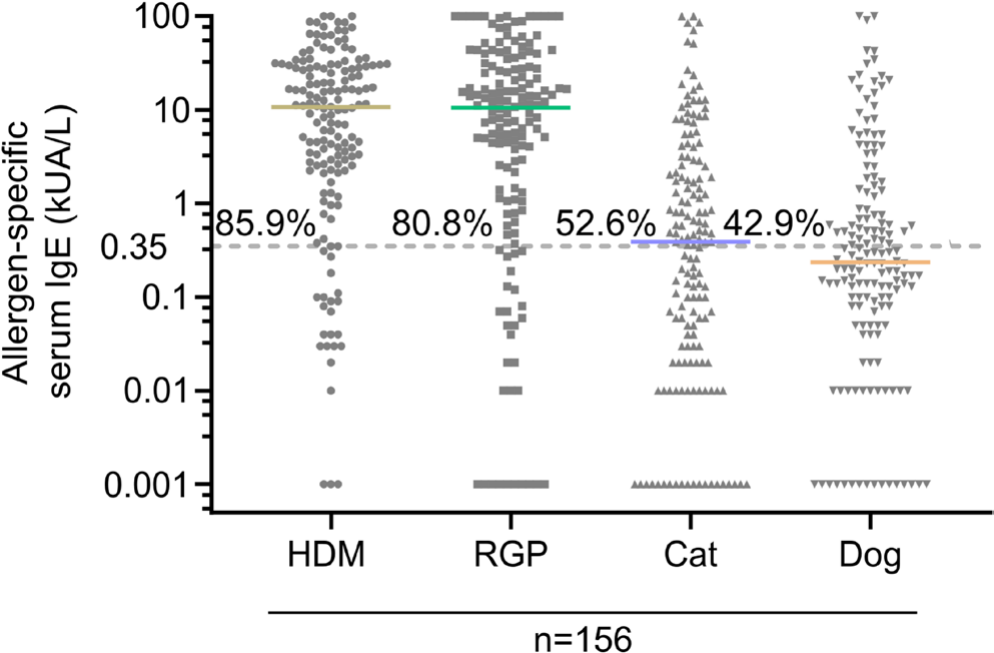
Allergen‐specific serum IgE results for HDM, RGP, cat and dog sensitization from the patient cohort. Serum‐specific IgE levels to HDM (gold), RGP (green), cat (purple) and dog (orange) of the allergic patient cohort (*n* = 156) as obtained by ImmunoCAP in the diagnostic work‐up. Individual data points are shown with a line at the median. For each allergen, the frequencies of patients with a positive serology (≥ 0.35 kUA/L; dotted line) are depicted.

A total of 156 patients (aged 17–74 years; 53.1% female) were included on the basis of having AR and/or asthma and sensitization to HDM, RGP, cat, and/or dog (Figure 1). Nearly all patients had AR (97.4%) and 78 (50%) individuals had asthma. Concomitant eczema was reported in 58 (37.2%) individuals, and 24.4% of patients had a coexistence of asthma, AR, and eczema. *N* = 35 (22.4%) patients were monosensitized, and *n* = 121 had 2 or more sensitizations (Table 1). Sensitization to HDM (*n* = 134; 85.9%) and RGP (*n* = 126; 80.8%) was more common than to cats (*n* = 82; 52.6%) or dogs (*n* = 67; 42.9%). Twenty‐one non‐atopic participants (22–54 years; 47.6% female) were recruited as the control group. The control subjects had no clinical history of AR or asthma and were not sensitized to HDM, RGP, cat, and dog (Table 1). Both cohorts were evenly split between males and females and had a similar age distribution (Table 1).

**TABLE 1:** Demographics of patient cohort with respiratory allergic diseases.

	Aeroallergic *n* = 156	Non‐atopic *n* = 21
Female sex *n* (%)	85 (53.1%)	10 (47.6%)
Median age (years; range)	31 (18–74)	28 (22–54)
Allergen sensitization, *n* (%)	156 (100%)	0 (0%)^a^
HDM	134 (85.9%)	0
RGP	126 (80.8%)	0
Cat	82 (52.6%)	0
Dog	67 (42.9%)	0
Monosensitization, *n* (%)	35 (22.4%)	0
Double sensitization, *n* (%)	42 (26.9%)	0
Triple sensitization, *n* (%)	26 (16.7%)	0
Quadruple sensitization, *n* (%)	53 (34.0%)	0
Respiratory allergic diseases	156 (100%)	0
Asthma *n* (%)	78 (50.0%)	0^b^
Allergic rhinitis (AR)	152 (97.4%)	0^b^
Perennial	105 (65.6%)	0
Seasonal	121 (77.6%)	0
Eczema	58 (37.2%)	0
Coexistence with both asthma and AR, *n* (%)	38 (24.4%)	0

Abbreviations: HDM, house dust mite; RGP, ryegrass pollen.

^a^ The subjects were defined as not sensitized to HDM, RGP, cat, and dog (sIgE < 0.35 kUA/L).

^b^ The subjects were included on the basis of the absence of any allergies.

### 3.2 AeroDiff CytoBas Panel Design and Optimization

**FIGURE 2:**
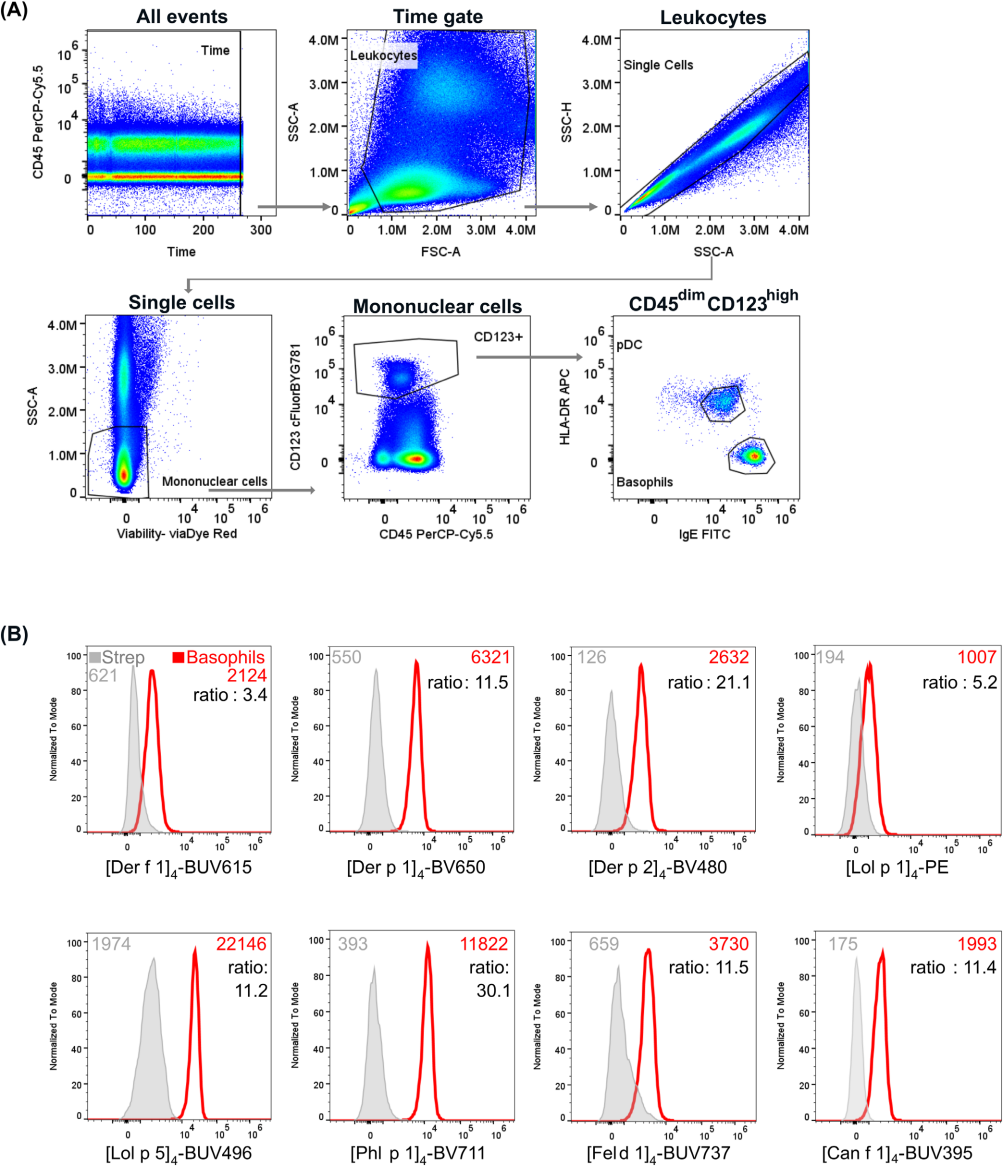
Multiplex CytoBas panel for aeroallergen sensitization detection on basophils from cryopreserved PBMC samples. (A) Stepwise gating strategy for the detection of basophils in PBMC samples. (B) Representative histograms depicting overlays of the streptavidin‐only control (strep; gray peak) with the allergen component tetramer staining on basophils from the same individual. In each plot, the median fluorescence intensities (MFI) are depicted, as well as the MFI ratio of allergen staining over strep control.

Eight major allergen components were successfully produced with 6‐His and BirA tags: Der f 1, Der p 1, Der p 2 (HDM), Lol p 1, Lol p 5, Phl p 1 (grass pollen), Fel d 1 (cat) and Can f 1 (dog) (Figure S1B). Each component was tetramerized with a distinct fluorochrome‐conjugated streptavidin, with the aim of minimizing spectral overlap, especially between components from the same allergen (Table S1). The panel was supplemented with a live/dead dye and 4 fluorochrome‐conjugated antibodies for accurate detection of blood basophils: CD45, CD123, IgE, and HLA‐DR on a 5‐laser spectral flow cytometer. The 13 fluorescent spectra were clearly resolved with minimal spectral overlap and had a complexity index of 3.46 (Figure S2). Second staining was performed on each sample containing the same antibodies and fluorochrome‐conjugated streptavidins only (no protein) as the negative control. The panel robustly resolved basophils (Figure 2A), and the staining intensity of each allergen component was determined through a ratio of MFI of the tetramer with the strep‐only control for each sample (Figure 2B). A ratio of ≥ 2 was defined as positive sensitization, as previously described [13, 24].

Upon completion of patient recruitment, an initial batch of coded samples was run and analyzed. Following unblinding, it was found that many fewer RGP allergic patients showed positive sensitization to Lol p 1, Phl p 1, and Lol p 5 with AeroDiff CytoBas than we had observed in a previous cohort [13]. Quality checks on these proteins revealed that the concentration of Lol p 1 was lower than initially presumed, and the biotinylation of Phl p 1 and Lol p 5 was suboptimal. Repeated analysis on remaining samples from the same patients with increased Lol p 1 protein and newly biotinylated batches of Phl p 1 and Lol p 5 showed increased staining intensities, whereas the other 5 components that were unchanged did not yield different results (Figure S3). All subsequent patient samples were analyzed with the optimized proteins, and only samples measured with the optimized conditions were included for further evaluation.

### 3.3 Distinct Differentiation of Sensitized and Non‐Sensitized Allergic Donors for Each Tetramer Component

**FIGURE 3:**
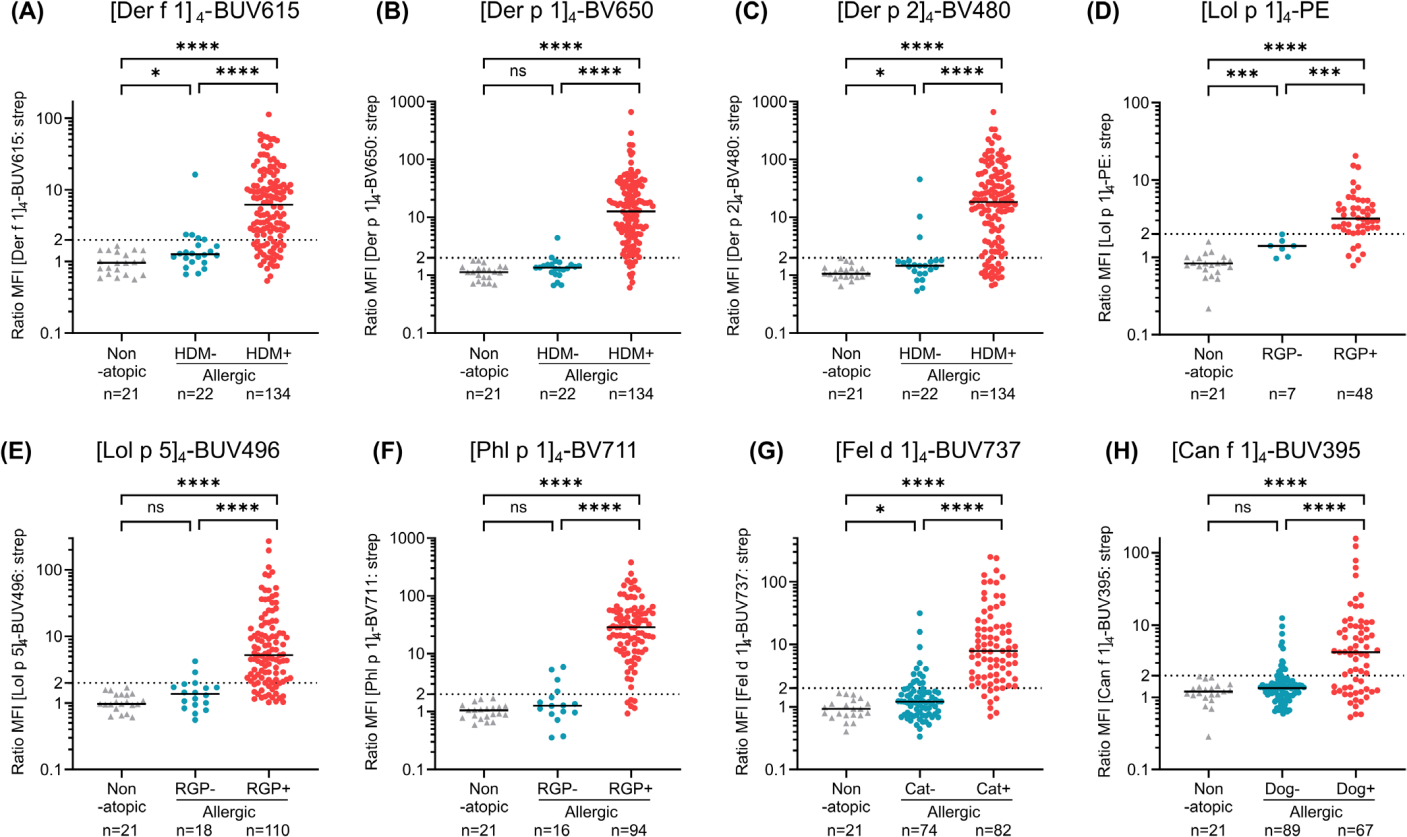
AeroDiff CytoBas staining intensities on basophils from non‐atopic, non‐sensitized, and sensitized individuals. Staining intensities (ratio MFI) of (A) [Der f 1]_4_‐BUV615, (B) [Der p 1]_4_‐BV650, (C) [Der p 2]_4_‐BV480, (D) [Lol p 1]_4_‐PE, (E) [Lol p 5]_4_‐BUV496, (F) [Phl p 1]_4_‐BV711, (G) [Fel d 1]_4_‐BUV737, and (H) [Can f 1]_4_‐BUV395 are plotted for comparison among non‐atopic and non‐sensitized, and sensitized allergic individuals. Statistics: Mann–Whitney *U* test; *p < 0.05; ***p < 0.001; ****p < 0.0001; ns, not significant.

For initial evaluation, patients were stratified based on allergic sensitization as determined by ImmunoCAP to HDM (for Der f 1, Der p 1 and Der p 2), RGP (for Phl p 1, Lol p 1 and Lol p 5), cat (for Fel d 1) and dog (for Can f 1). Thus, AeroDiff CytoBas results for each component were compared between 3 groups: non‐atopic control, and patients with and without sensitization to that allergen (Figure 3). The MFI ratios for all non‐atopic controls were below 2 for all eight components. Similarly, the MFI ratios for allergen components were < 2 for nearly all of the non‐sensitized patients. In contrast, the MFI ratios were much higher for all eight components in the sensitized patients, and these were highly significant (*p* < 0.0001) for all components against both the non‐atopic and the non‐sensitized groups (Figure 3).

### 3.4 Sensitivity and Specificity of CytoBas Compared to Serology for Allergen Sensitization Detection

**FIGURE 4:**
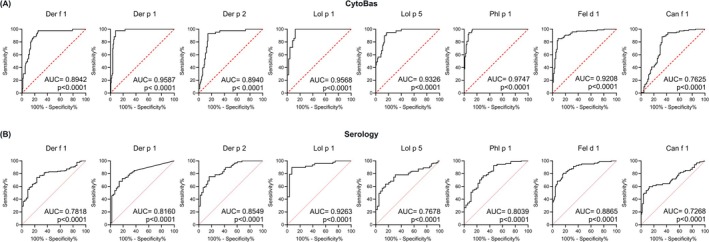
Evaluation of diagnostic performance of CytoBas and IgE serology for each aeroallergen component. (A) ROC analysis based on staining intensity as determined by CytoBas, and (B) as determined by IgE serology of [Der f 1]_4_‐BUV615, [Der p 1]_4_‐BV650 and [Der p 2]_4_‐BV480 for HDM sensitization, of [Lol p 1]_4_‐PE, [Lol p 5]_4_‐BUV496 and [Phl p 1]_4_‐BV711 for ryegrass pollen sensitization, of [Fel d 1]_4_‐BUV737 for cat, and [Can f 1]_4_‐BUV395 for dog sensitization. The areas under the curve (AUC) and the significance are depicted.

The diagnostic performance of the AeroDiff CytoBas was evaluated using receiver‐operating characteristic (ROC) analysis. Patients were stratified based on the ImmunoCAP results, and the non‐sensitized allergic and non‐atopic donors were combined into the control group for comparison with the sensitized allergic donors (Figure S4). Each component individually separated the controls from the sensitized patients with high significance (*p* < 0.0001). The AUCs ranged from 0.89–0.97, except for Can f 1, which had an AUC of 0.76 (Figure 4).

**FIGURE 5:**
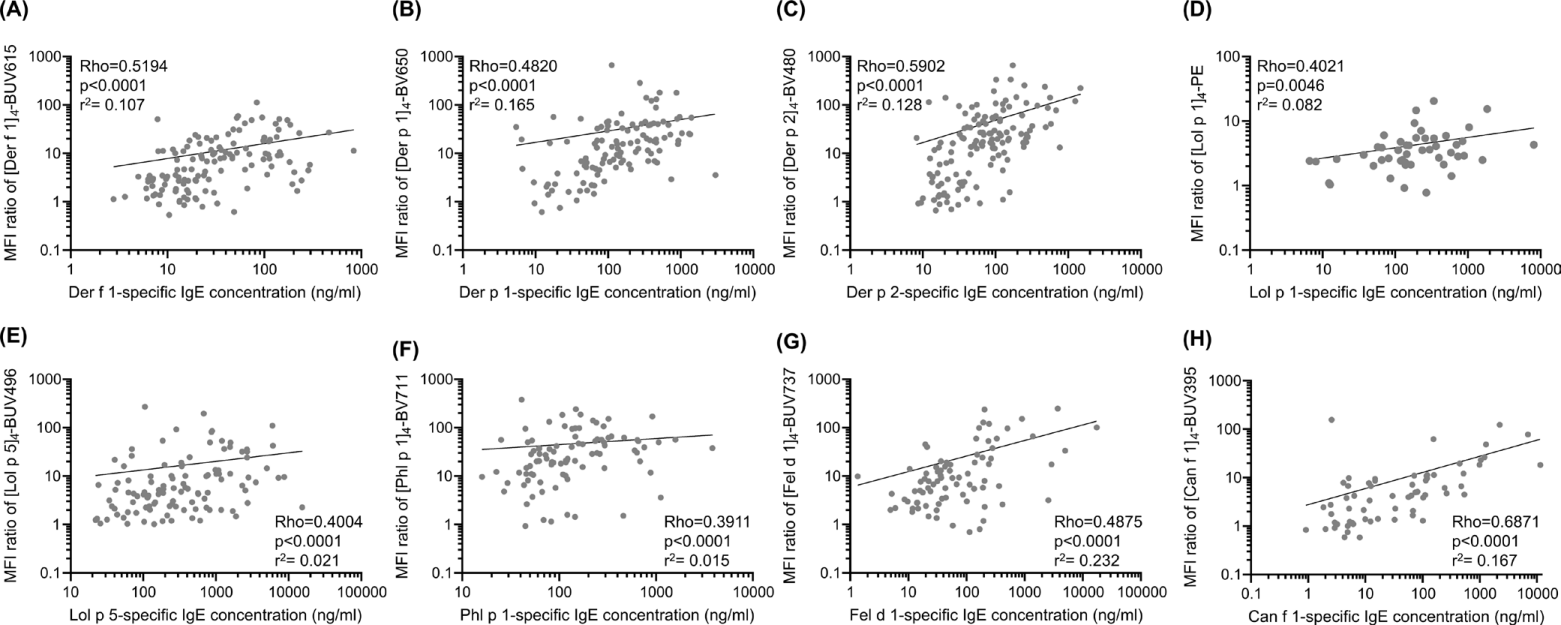
Correlation between specific IgE binding intensities on basophils and serum‐specific IgE concentrations. Correlation plots between circulating specific IgE levels and basophil tetramer binding staining intensities for all assessed allergic individuals for (A) [Der f 1]_4_‐BUV615, (B) [Der p 1]_4_‐BV650, (C) [Der p 2]_4_‐BV480, (D) [Lol p 1]_4_‐PE, (E) [Lol p 5]_4_‐BUV496, (F) [Phl p 1]_4_‐BV711, (G) [Fel d 1]_4_‐BUV737, and (H) [Can f 1]_4_‐BUV395. Individual data points are shown with log–log regression lines. Spearman's rank correlation tests were conducted, with the rho correlation coefficient indicating the monotonic relationship between the CytoBas and serology tests. The *p*‐values indicate the significance of the correlation tests. The *r*‐squared (*r* ^2^) values are presented to indicate the strength of the regression relationship.

CytoBas uses individual allergen components, whereas the clinical diagnosis was performed with allergen extracts. Therefore, we quantified for each study participant the serum‐specific IgE levels to the same recombinant allergen components that were included in the CytoBas assay (Figure S4B). Similar to CytoBas, the component serology separated the controls from the sensitized patients with high significance (*p* < 0.0001) using ROC curve analysis (Figure 4B). Still, for each component, the AUC of serology was smaller than that of the CytoBas. Importantly, the diagnostic capacity of CytoBas was significantly greater for Der f 1, Der p 1, Lol p 5, and Phl p 1 (bolded in Table S2). To evaluate whether the basophil‐bound IgE levels as determined by CytoBas were related to the serum IgE levels, we plotted the specific IgE levels against the tetramer staining (ratio MFI) (Figure 5). The correlation analysis indicated moderate to strong positive correlations with significance (*p* < 0.0001) for all components, with Der p 2 and Can f 1 showing higher correlation coefficients (rho > 0.6) than the other 6 components (Table S2). However, the r‐squared values were low to minimal (*r* ^2^ < 0.25). This might indicate that the cell‐bound IgE as quantified by CytoBas comprises a different pool of IgE that is not reflected by free serum IgE.

### 3.5 Evaluation of Cross‐Reactivity Between Homologous Allergens With CytoBas

Globally, there are two predominant HDM species with distinct and overlapping geographical distribution patterns. In South‐East Australia, *D. pteronyssinus* is the major sensitizer, as *D. farinae* is virtually absent. Although group 1 HDM components show high homology, the level of cross‐reactivity remains unresolved, e.g., how well Der f 1 is recognized in Der p 1 sensitized individuals. Our multiplex CytoBas included both Der f 1 and Der p 1, and their positive staining showed moderate rank correlation (Spearman's rho coefficient of 0.57), which was higher than that between Der f 1 and Der p 2 (rho = 0.15) (Table S3). To understand how well the cross‐reactivity of Der f 1 and Der p 1 allergen can be predicted in CytoBas, the ratio MFI correlation between [Der f 1]_4_‐BUV615 and [Der p 1]_4_‐BV650 was plotted as a regression line, showing a weak relationship (*r* ^2^ = 0.1685) (Figure S5). Detection with single Der f 1 in CytoBas provided a much lower sensitivity (78.4%) and poorer overall diagnostic performance (ROC 0.8972) than that of Der p 1 (91.8%; ROC 0.9587) (Table S3).

In parallel, sensitization was evaluated to Phl p 1, a major allergen from Timothy grass pollen, which is widespread in Europe, whereas the related ryegrass is the major sensitizer for seasonal asthma and AR in southern Australia. The homologous allergen components Phl p 1 and Lol p 1 showed a high correlation in positive sensitization detection (Spearman's rho coefficient 0.795) and a moderate predictive ability (*r* ^2^ = 0.4878), in contrast to Phl p 1 and Lol p 5 showing no correlation (rho = 0.076) (Table S3; Figure S5). While the diagnostic performance of Phl p 1 was comparable to Lol p 1 (0.9568 vs. 0.9747), Phl p 1 yielded false positives (Figure S4A), providing a lower specificity than detection with Lol p 1 in CytoBas.

Overall, the findings suggested a moderate cross‐reactivity detected via CytoBas between the homologous allergens, Der f 1 and Der p 1 sensitization among HDM‐sensitized individuals, as well as Lol p 1 and Phl p 1 among GP‐sensitized individuals. On the other hand, it underscored the importance of including geographically relevant components, such as Der p 1 or Lol p 1 for Australia and New Zealand.

### 3.6 Improved Diagnostic Performance of CytoBas With Two Major Components of the Same Allergen

**FIGURE 6:**
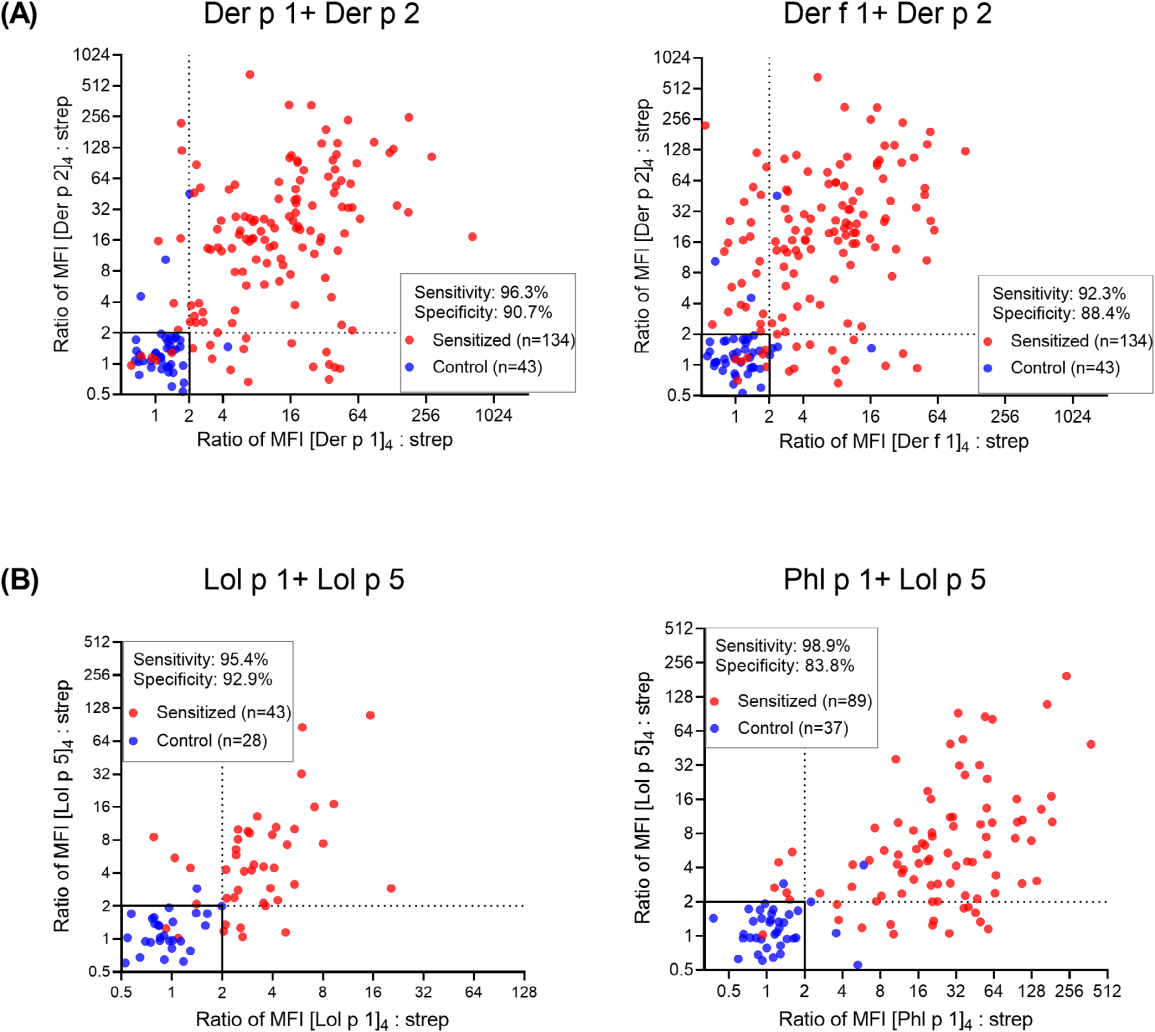
Combined evaluation of two allergen components for detection of HDM or ryegrass pollen sensitization. (A) Scatterplots depicting MFI ratio of allergen: Streptavidin of [Der p 1]_4_‐BV650 versus [Der p 2]_4_‐BV480 and of [Der f 1]_4_‐BUV615 versus [Der p 2]_4_‐BV480 on basophils from HDM‐sensitized and unsensitized donors. (B) Scatterplots depicting MFI ratio of allergen: Streptavidin of [Lol p 1]_4_‐PE versus [Lol p 5]_4_‐BUV496 and of [Phl p 1]_4_‐BV711 versus [Lol p 5]_4_‐BUV496 on basophils from grass pollen‐sensitized and unsensitized donors. The depicted sensitivity and specificity values are based on positivity of one or both components. Cutoff values at the ratio of 2 are depicted by dotted lines.

The AeroDiff CytoBas assay included two major allergen components for both HDM and RGP. As both components are evaluated in a single assay, there is the potential for a combined analysis with a potentially improved diagnostic performance. When HDM sensitization is defined as a positive result of Der p 1 and/or Der p 2 (Figure 6A), this improves the sensitivity over single component analysis (Der p 1: 91.8%; Der p 2: 85.3%) with minimal impact on specificity (Table S4), providing high sensitivity (96.3%) and specificity (90.7%) for HDM allergy diagnosis. Despite the high homology between Der f 1 and Der p 1, combined analysis of Der f 1 and Der p 2 provided a lower sensitivity (94.8%) and specificity (83.7%) (Figure 6A; Table S4) than the Der p 1 and Der p 2 combination.

Combined evaluation of Lol p 1 and Lol p 5 for RGP sensitization yielded a 95.4% sensitivity and 92.9% specificity (Figure 6B), effectively increasing the sensitivity over single component use (Lol p 1: 87.5%; Lol p 5: 82.7%). When substituting Lol p 1 for Phl p 1, the combined Phl p 1 and Lol p 5 analysis yielded a higher sensitivity (98.9%), but reduced specificity (86.5%). Thus, the inclusion of two major RGP components from the same allergen in a single CytoBas improves the diagnostic accuracy for allergen sensitization.

## 4 Discussion

We here present a multiplex flow cytometry single assay approach for accurate detection of HDM, grass pollen, cat, and dog allergen sensitization in Australian patients with allergic rhinitis and/or asthma. Cytometric detection of allergen component binding on basophils (CytoBas) showed diagnostic performance similar to or greater than IgE serology for each of the eight included components: Der f 1, Der p 1, Der p 2, Lol p 1, Lol p 5, Fel d 1, and Can f 1. Detection of sensitization to either one or two components from the same allergen generated improved diagnostic performance for both HDM (Der p 1 and Der p 2) and RGP (Lol p 1 and Lol p 5). Thus, tailored multiplex cytometric approaches, such as an AeroDiff CytoBas, provide rapid and accurate component‐resolved diagnosis based on detection of functional allergen‐specific IgE on the surface of basophils.

This current study included a non‐allergic control group as well as prospectively recruited patients with AR and/or asthma, irrespective of allergen sensitization. Thus, in contrast to our previous studies [13, 24], we could evaluate the performance of CytoBas in the relevant target population, that is, symptomatic patients with versus without the relevant allergen sensitization. Patients were stratified on the basis of serum‐specific IgE to allergen extracts, that is, standard clinical practice at our institution. This is presumed to be more accurate than skin prick testing, as variability in the allergen extract used and patient‐specific skin conditions may lead to inconsistent results [47, 48]. Although recruited from a single site, the clinical characteristics of our adult cohort were similar to reports from other sites, with frequent concomitant asthma and AR [49, 50], and a lower prevalence of eczema [49, 51, 52]. Importantly, the sensitization profiles were consistent with previous studies, reporting greater prevalence of sensitization to HDM and RGP than to pets [37, 38, 39]. Almost 80% of patients in our cohort were polysensitized to two or more of the four allergens (HDM, RGP, cat and dog). This is in line with previous studies that reported polysensitization rates of 64%–80% [5, 53, 54, 55].

Our results showed that both component‐resolved approaches for allergen sensitization, the CytoBas and serum‐specific IgE ELISA, exhibited high diagnostic accuracies (ROC AUC: 0.73–0.97). There were false positives and false negatives when compared to the gold standard ImmunCAP with allergen extracts—although this gold standard does have its own limitations [56]. Allergen extracts, particularly for dog, can contain inconsistent or low levels of the major allergens, e.g. Can f 1, which can lead to reduced detection sensitivity in ImmunoCAP [57]. In our cohort, ~20% of allergic patients within the non‐sensitized group were detected as false positives showing positive staining via CytoBas or specific IgE binding to cat (Fel d 1) and dog (Can f 1) components; however, all non‐atopic individuals remained negative. This suggests the CytoBas approach may be more sensitive in detecting pet allergen sensitization than the standard ImmunoCAP using extracts [33, 58].

On the other hand, false negatives were also observed for both CytoBas and specific serology assays for individual components. While this can be a test error, it might also indicate that those patients were not sensitized to that particular allergen component. This limitation can be overcome by including more than one immunodominant allergen for detecting a particular allergy, as we demonstrated by the HDM or grass pollen sensitization detection with two components in multiplex CytoBas. For cat allergy, Fel d 1 is the most prevalent allergen (~90% of patients), with an estimated 50% of patients being monosensitized to Fel d 1 [59, 60]. Cohort studies in various regions found that 30%–50% of allergic subjects were sensitized to Fel d 4 and/or Fel d 7 [61, 62]. Fel d 4 and Fel d 7 belong to the lipocalin protein group different from the secretoglobin family of Fel d 1, which may provide additional insight for patient allergy management [63]. Furthermore, sensitization to Fel d 4 was suggested to be independently associated with increased type‐2 inflammation [64] and severe asthma in patients [65]. Therefore, the inclusion of Fel d 4 and/or Fel d 7, alongside Fel d 1, could enhance diagnostic accuracy for cat allergy in AeroDiff CytoBas.

For the detection of dog allergen sensitization, recent studies have suggested that the inclusion of other components on top of Can f 1, such as Can f 2, Can f 4, and Can f 5, can improve diagnosis and assist with the prediction of asthma risk [66, 67]. A recent study among Swedish adults reported sensitization to Can f 1 as the major sensitizer (43%) and Can f 5 as the second‐most prevalent (33%) allergen [61]. Can f 5 is a prostatic kallikrein protein that is found only in male dogs, and it was the most common allergen for patients sensitized to dogs only, but not cats [68, 69]. Some studies from Asia and Europe reported various degrees of monosensitization to Can f 5 (4%–58%), and its sensitization was relevant to persistent AR [61, 65, 70]. Can f 2 and Can f 4 belong to the same lipocalin family as Can f 1 [30]. Sensitization to Can f 2, which occurs commonly as co‐sensitization to Can f 1, was reported to be associated with severe asthma in children [71]. Among dog‐allergic patients, 35%–46% had IgE reactivity to Can f 4 [72]. Taken together, the inclusion of at least Can f 5 and potentially Can f 2 and Can f 4, in addition to Can f 1, could considerably increase the diagnostic sensitivity for dog allergen sensitization detection with CytoBas.

There are some technical considerations to be addressed before implementing CytoBas as a flow cytometry‐based diagnostic in clinical practice. We here experienced that recombinant protein stability was affected, especially when concentrations were < 1 mg/mL, likely due to denaturing or degradation [73]. Additionally, protein batch consistency was limited by low‐scale in‐house production. Large‐scale recombinant protein production under standard GMP could ensure protein quality and batch consistency for recombinant proteins [74]. More allergen tetramers can be added to the panel, which is only restricted by commercially available streptavidin fluorochromes for conjugation and the multichannel capacity on the spectral flow cytometer [75]. Furthermore, the basophil population in our thawed PBMC (0.2%–0.35%) was smaller than we had observed previously in fresh whole blood (1%–2% of mononuclear cells). As a result, the observations from this fraction potentially do not represent the total basophil population. The loss of basophils could be due to Ficoll separation and/or freeze‐thawing, which could preferentially lead to loss of basophils rich in granules. In previous studies, we performed basophil staining on fresh basophils (incl Lol p 1, Der f 1, Der p 1 and Der p 2) and these showed a homogeneous staining pattern [13, 24]. In addition, we showed that basophil binding did not differ between degranulated versus non‐degranulated basophils after activation [13, 24]. Thus, we are confident that the analysis in the current study accurately measured sensitization. We do recommend that, similar to our approach, in further studies with additional allergen components, the CytoBas approach will be tested in parallel on fresh whole blood and thawed PBMC with at least 5000 basophil events per analysis. This is to ensure that the thawed fraction is representative of the whole basophil population.

Multiplex CRD assays are informative for the differential diagnosis and management of AR and asthma [9, 76], and particularly valuable for patients with polysensitization who are at greater risk of developing more severe asthma symptoms [77]. Serum‐specific IgE multiplex CRD assays are currently available and can provide extensive panels with up to 300 allergens and components [9]. However, several groups have reported limitations with these assays, identifying a lack of flexibility or relevance of many allergens in various cohorts, which can sometimes cloud rather than clarify the clinical picture [78, 79]. Our more focused approach with CytoBas evaluating clinically relevant allergens and components could be more cost‐effective and informative.

Our AeroDiff CytoBas panel was tailored for clinically relevant detection of aeroallergies in south‐eastern Australia. The composition can be tailored to the specific needs of other geographical regions. For the detection of HDM sensitization, Der p 23 has lately been considered essential by many research groups [80, 81]. Across different studies, 5%–8% of HDM‐allergic patients are monosensitized to Der p 23 [82, 83]. In addition, sensitization to Der p 23 is associated with worse clinical symptoms in patients suffering from moderate to severe asthma [84, 85]. Furthermore, other grass, tree, and weed pollen components can be considered. Pas n 1 from Bahia grass [86] and Cyn d 1 from Bermuda grass [87] could be relevant for subtropical regions, such as northern Australia [88]. In Europe, birch trees are common, and the major allergen, Bet v 1, showed similarly high sensitization as Phl p 1, Der p 2, and Fel d 1 in a German allergic cohort (*n* = 500) [89]. Finally, mold allergy can be considered, which was found prevalent among respiratory‐allergic individuals from the USA [90]. The addition of more discerning components, such as (Alt a 1) from mold and Bet v 1 to our current CytoBas panel, will provide relevance to European and US patients.

Our results showed that detection with Der f 1 demonstrated a lower sensitivity than Der p 1 for HDM allergy, suggesting that it is important to include the primary sensitizer (Der p 1) in the AeoDiff CytoBas analysis. As *D. farinae* is more prevalent in Asia and the USA [34], it would be important to evaluate Der f 1 in these regions, perhaps initially in parallel to Der p 1 to determine whether the primary sensitizer is more sensitive. Similarly, the inclusion of Phl p 1 for the evaluation of European patients is important as Timothy grass is more common there than ryegrass. In summary, the inclusion of homologous proteins should be evaluated on a case‐by‐case basis and considered if it improves diagnostic performance.

The BAT is considered to provide better detection of clinically relevant sensitization [23, 90], as serum IgE does not necessarily determine the clinical manifestations of allergic diseases [91]. We employed the CytoBas assay to directly detect basophil‐bound specific IgE and compared these results with traditional serum specific IgE measurements. A minimal to moderate correlation between the two approaches (Spearman's rho 0.4–0.68) was found, suggesting partial concordance. However, the weak log–log regression and low r‐squared values indicate that CytoBas does not capture all variability in serum IgE levels, indicating that cell‐bound IgE and serum IgE are two distinct reservoirs. In addition, studies showed that serum IgE levels were not correlated well with the severity or reaction threshold of food allergy [92]. Therefore, CytoBas can potentially be developed as a non‐invasive and efficient diagnostic tool for these allergies, especially peanut and tree nut allergies, as some of the most concerning elicitors of life‐threatening anaphylaxis [93].

Taken together, this study has demonstrated the capability of CytoBas in multiplex CRD for aeroallergen sensitization, with greater sensitivity and specificity than component‐specific IgE serology. AeroDiff CytoBas will be particularly beneficial in understanding sensitization profiles tailored to specific populations or individuals so that it can inform effective AIT prescriptions for patients suffering from respiratory allergies [6]. Furthermore, CytoBas can be translated for other severe allergies to enhance clinical decision‐making for allergy management.

## Author Contributions

L.H. and P.M.A. measured and interpreted the data; M.H. and K.D. included patients and performed clinical measurements; M.H., R.E.O'H., and M.C.v.Z. provided funding, designed the study, and interpreted the data; and L.H. and M.C.v.Z. wrote the manuscript. P.M.H., P.M.A., K.D., M.H., and R.E.O'H. edited the manuscript draft.

## Conflicts of Interest

M.C.v.Z., R.E.O'H., and P.M.H. are inventors on a patent related to this work (PCT/AU2020/051116). All other authors declare no conflicts of interest.

## Supporting information


**Data S1.** Supplementary Figures and Tables.

## Data Availability

The data that support the findings of this study are available from the corresponding author upon reasonable request.
