# Supplementary material for: A Single Multiplex CytoBas Assay Incorporating Eight Major Components for Accurate Detection of Allergen Sensitization in Asthma and Allergic Rhinitis
Source: Allergy. 2025 Mar 7;80(4):1047–59. doi: 10.1111/all.16513 (PMC11969309; doi:10.1111/all.16513)
Supplement: Supplementary file 1 — Data S1. Supplementary Figures and Tables. [file ALL-80-1047-s001.docx]

## **Supplementary Tables** (n=4)

#### **Supplementary Table 1. Differential diagnosis of aeroallergen sensitization (AeroDiff CytoBas) panel components and antibody list.**

| **Allergen tetramer** | **Reagent** | **Fluorochrome** | **Clone** | **Vendor** | **Cat. Number** | **Volume/ test (μl)** |
| --- | --- | --- | --- | --- | --- | --- |
| [Der f 1]_4_ | Streptavidin | BUV615 | - | BD Biosciences | 613013 | 3.7 |
| [Der p 1]_4_ | Streptavidin | BV650 | - | Biolegend | 405232 | 0.7 |
| [Der p 2]_4_ | Streptavidin | BUV395 | - | BD Biosciences | 564176 | 6.5 |
| [Lol p 1]_4_ | Streptavidin | BV480 | - | BD Biosciences | 564876 | 0.8 |
| [Lol p 5]_4_ | Streptavidin | BUV496 | - | BD Biosciences | 564666 | 3.9 |
| [Phl p 1]_4_ | Streptavidin | BV711 | - | BD Biosciences | 563262 | 4.2 |
| [Fel d 1]_4_ | Streptavidin | BUV737 | - | BD Biosciences | 564293 | 6.6 |
| [Can f 1]_4_ | Streptavidin | R-PE | - | Life Technologies | S866 | 6.3 |
| - | CD45 | PerCP-Cy5.5 | 2D1 | BD Biosciences | 340953 | 2 |
| - | CD123 | cFluor BYG781 | SK3 | Cytek Biosciences | R7-11004 | 0.1 |
| - | IgE | FITC | Goat anti-human polyclonal | Life Technologies | H15701 | 1.25 |
| - | HLA-DR | APC | L243 | Biolegend | 307610 | 1 |
| - | Viability dye | viaDye Red | - | Cytek Biosciences | R7-60008 | 0.025 |

#### **Supplementary Table 2. Summary of ROC analysis and correlation of CytoBas staining to serum specific IgE levels for each allergen component**

| **Component** | **Allergic** | **Non-sensitized+ non-atopic** | **AUC from CytoBas ROC** | **AUC from for IgE serology ROC** | **Differences in ROC AUC between CytoBas and IgE serology** | **Correlation of CytoBas to serology** |
| --- | --- | --- | --- | --- | --- | --- |
| Der f 1 | 134 | 43 | **0.8942**^†^ | 0.7818 | p= 0.009 | Rho=0.5194 p<0.0001  r^2^= 0.107 |
| Der p 1 | 134 | 43 | **0.9587**^†^ | 0.8160 | p< 0.001 | Rho=0.4934, p<0.0001  r^2^= 0.03 |
| Der p 2 | 134 | 43 | 0.8940 | 0.8549 | p= 0.288 | Rho=0.5902, p<0.0001  r^2^= 0.128 |
| Lol p 1 | 48 | 28 | 0.9568 | 0.9263 | p= 0.410 | Rho=0.4021, p=0.0046  r^2^= 0.082 |
| Lol p 5 | 110 | 39 | **0.9326**^†^ | 0.7678 | p< 0.001 | Rho=0.4004, p<0.0001  r^2^= 0.021 |
| Phl p 1 | 94 | 37 | **0.9747**^†^ | 0.8039 | p< 0.001 | Rho=0.3911, p<0.0001  r^2^= 0.015 |
| Fel d 1 | 82 | 95 | 0.9208 | 0.8865 | p= 0.317 | Rho=0.4875, p<0.0001  r^2^= 0.232 |
| Can f 1 | 67 | 110 | 0.7625 | 0.7268 | p= 0.524 | Rho=0.6871, p<0.0001  r^2^= 0.167 |
| ^†^Significantly higher than the AUC from IgE serology (nonparametric DeLong test). | | | | | | |

**Supplementary Table 3. Spearman correlation of CytoBas staining of HDM and RGP allergens of the sensitized group.**

| **Allergy** | **Spearman correlation among allergen components^†^** |
| --- | --- |
| HDM | Der p 1 to Der p 2: Rho= 0.268; p=0.002  Der p 1 to Der f 1 Rho= 0.569; **p<0.0001**  Der p 2 to Der f 1 Rho= 0.150; p=0.084 |
| RGP | Lol p 1 to Lol p 5: Rho= 0.186; p=0.232  Lol p 1 to Phl p 1: Rho= 0.795; **p<0.0001**  Lol p 5 to Phl p 1: Rho= 0.076; p=0.630 |
| ^†^Correlation calculated based on normalized basophil staining ratio MFI values. | |

**Supplementary Table 4. Diagnostic performance of CytoBas assays with HDM and RGP allergen components**

| **Component** | **True**  **Positive** | | **False**  **Positive** | **False**  **Negative** | **True**  **Negative** | **Sensitivity (%)** | **Specificity (%)** | | **Accuracy (%)** |
| --- | --- | --- | --- | --- | --- | --- | --- | --- | --- |
| Both Der p 1 Der p 2  134 allergic 43 non-sens+non-atopic | | 129 | 4 | 5 | 39 | **96.3** | **90.7** | **94.9** | |
| Both Der f 1 Der p 2  134 allergic 43 non-sens+non-atopic | | 127 | 7 | 7 | 36 | **94.8** | **83.7** | **92.1** | |
| Both Der p 1 Der f 1  134 allergic 43 non-sens+non-atopic | | 132 | 5 | 11 | 38 | **92.3** | **88.4** | **91.4** | |
| Der f 1 only  134 allergic 43 non-sens+non-atopic | | 105 | 5 | 29 | 38 | **78.4** | **88.4** | **80.8** | |
| Der p 1 only  134 allergic 43 non-sens+non-atopic | | 123 | 2 | 11 | 41 | **91.8** | **95.4** | **92.7** | |
| Der p 2 only  134 allergic 43 non-sens+non-atopic | | 115 | 3 | 19 | 40 | **85.3** | **93.0** | **87.6** | |
| Both Lol p 1 and Lol p 5  43 allergic 28 non-sens+non-atopic | | 41 | 1 | 2 | 27 | **95.4** | **96.4** | **95.8** | |
| Both Phl p 1 and Lol p 5  89 allergic 37 non-sens+non-atopic | | 88 | 5 | 1 | 32 | **98.9** | **86.5** | **95.2** | |
| Both Lol p 1 and Phl p 1  48 allergic 37 non-sens+non-atopic | | 44 | 1 | 4 | 36 | **91.7** | **97.3** | **94.1** | |
| Lol p 1 only  43 allergic 28 non-sens+non-atopic | | 37 | 0 | 6 | 28 | **86.0** | **100** | **91.6** | |
| Lol p 5 only  43 allergic 28 non-sens+non-atopic | | 33 | 1 | 7 | 27 | **82.5** | **96.4** | **88.2** | |
| Phl p 1 only  48 allergic 37 non-sens+non-atopic | | 45 | 1 | 3 | 36 | **93.7** | **97.3** | **95.3** | |

# **Supplementary Figures** (n=5)

*
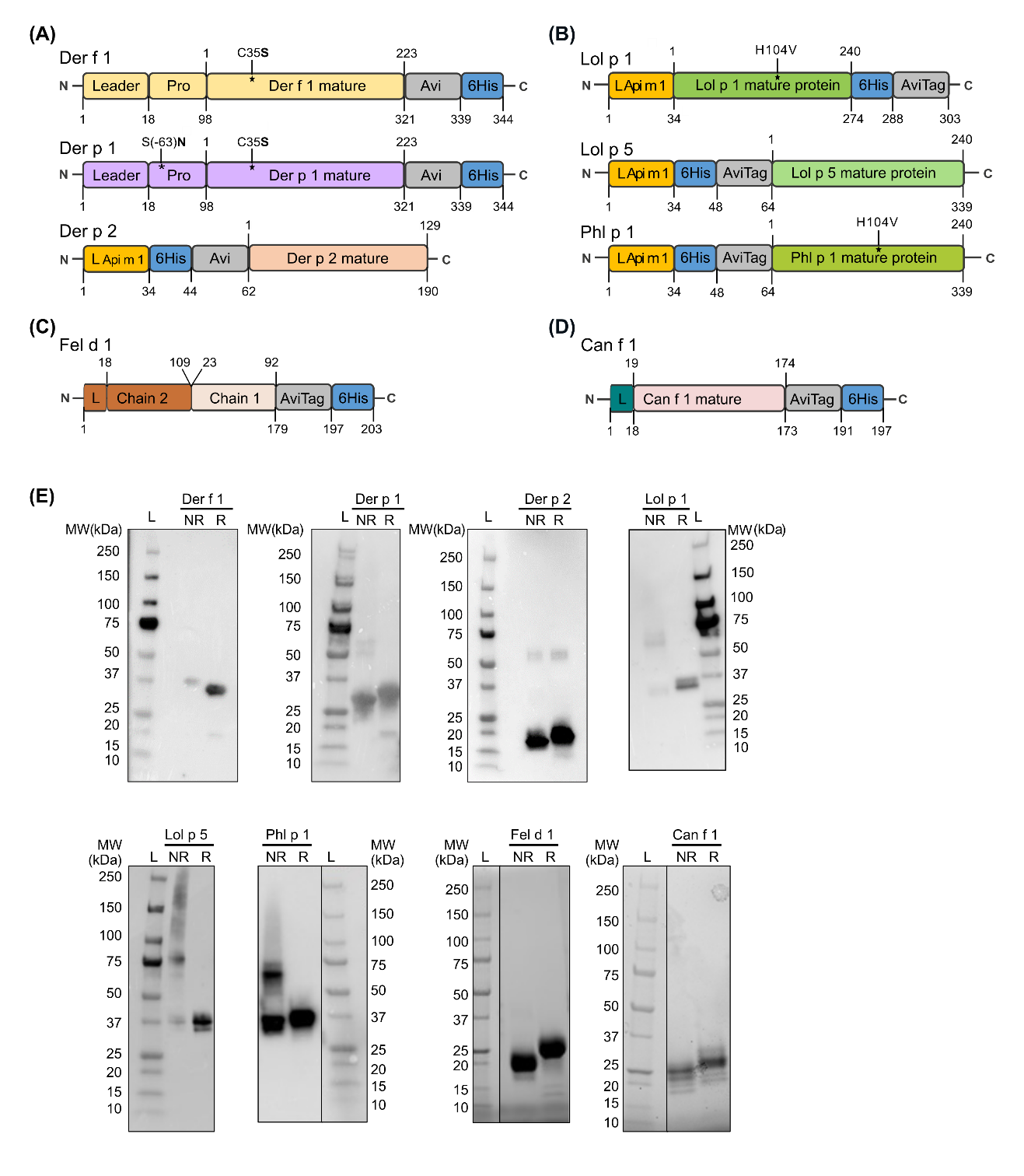
*

Supplementary Figure 1. Detection of allergen sensitization by quantifying binding of fluorescent components of aeroallergens to basophils. Schematic diagram of DNA constructs for **(A)** HDM components, Der f 1, Der p 1 and Der p 2, **(B)** grass pollen components, Lol p 1, Lol p 5 and Phl p 1, **(C)** cat dander component Fel d 1, and **(D)** dog dander component Can f 1 for protein generation. **(E)** Anti-His western blots of recombinant allergens loaded under non-reduced (NR) and reduced (R) conditions. Protein ladders (L) were run alongside to assess the molecular weight (MW). Sequence-predicted sizes: Der f 1 (37 kDa); Der p 1 (37 kDa); Der p 2 (21 kDa); Lol p 1 (33 kDa); Lol p 5 (21 kDa); Phl p 1 (33 kDa); Fel d 1 (21 kDa); Can f 1 (37 kDa).


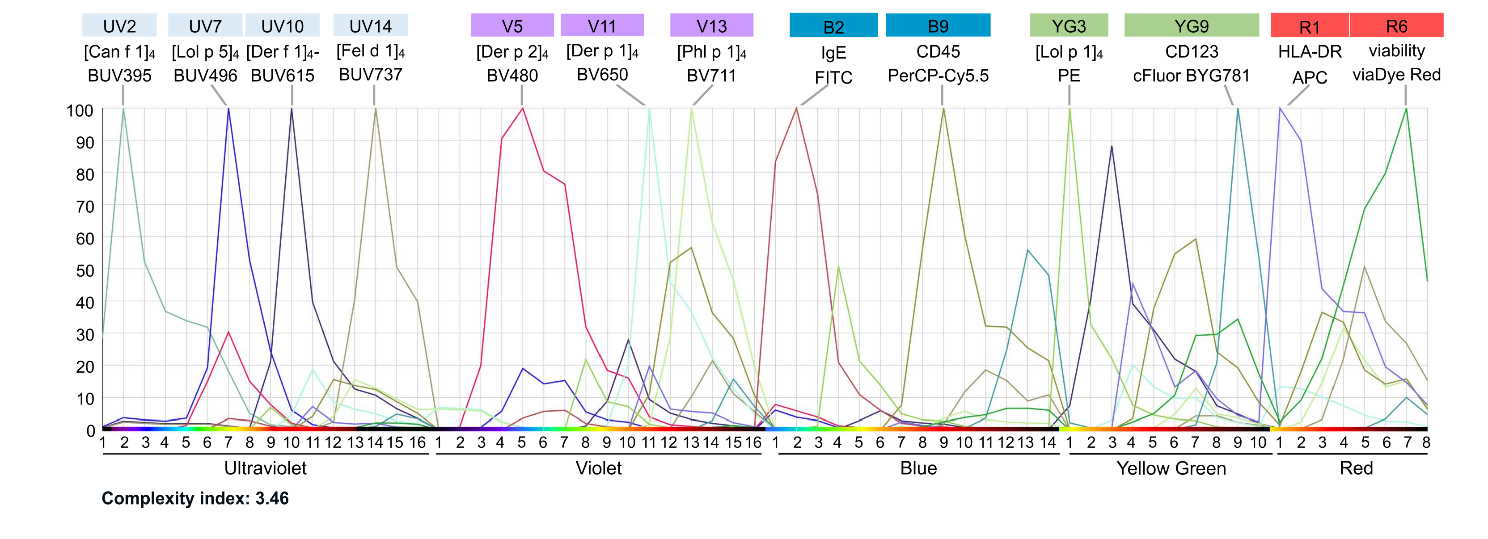
Supplementary Figure 2. Optimization of multiplex CytoBas panel for aeroallergen sensitization detection on basophils from cryopreserved PBMC. Spectral overview of the CytoBas panel for the 5-laser Cytek Aurora^TM^. For each emission spectrum, the fluorescent marker is depicted, as well as the spectral channel with the peak emission. Fluorochromes were chosen with the aim to minimize interference for these 13 markers. Complexity index (3.46) was calculated on the Cytek Cloud.

Supplementary
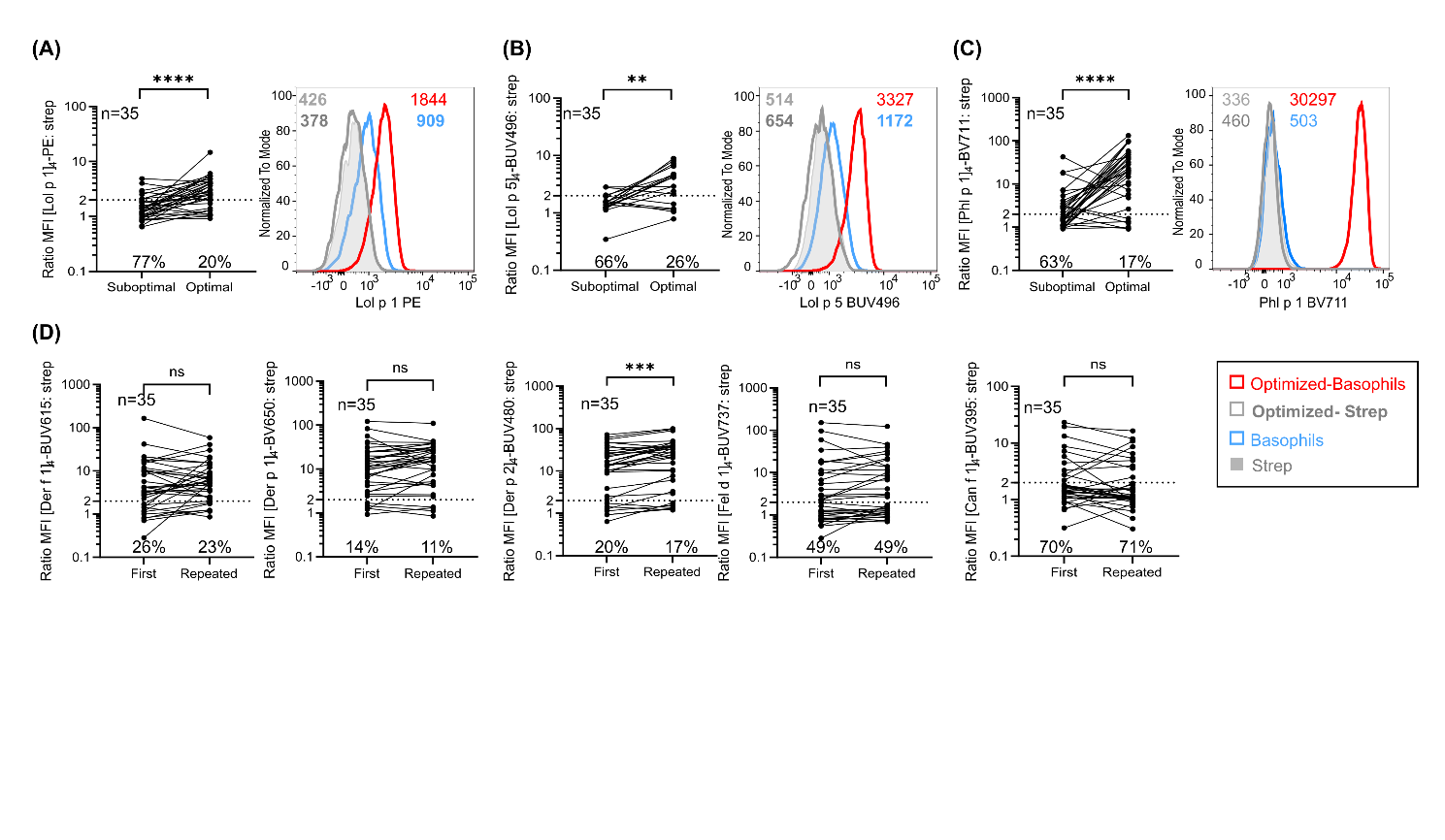
Figure 3. Technical optimization of allergen tetramers for detection of allergen sensitization with multiplex CytoBas. Example histograms and paired results are shown for initial results (blue) and repeat measurements (red) using adjusted allergen tetramers for (**A**) [Lol p 1]_4_-PE, (**B**) [Lol p 5]_4_-BUV496, and (**C**) [Phl p 1]_4_-BV711. (**D**) Pair-wise comparison in staining intensity of repeat measurements with the unchanged [Der f 1]_4_-BUV615, [Der p 1]_4_-BV650, [Der p 2]_4_-BV480, [Fel d 1]_4_-BUV737 and [Can f 1]_4_-BUV395. Statistics: Wilcoxon signed-rank test. **p < 0.01, ***p < 0.001, ****p < 0.0001, ns: not significant.


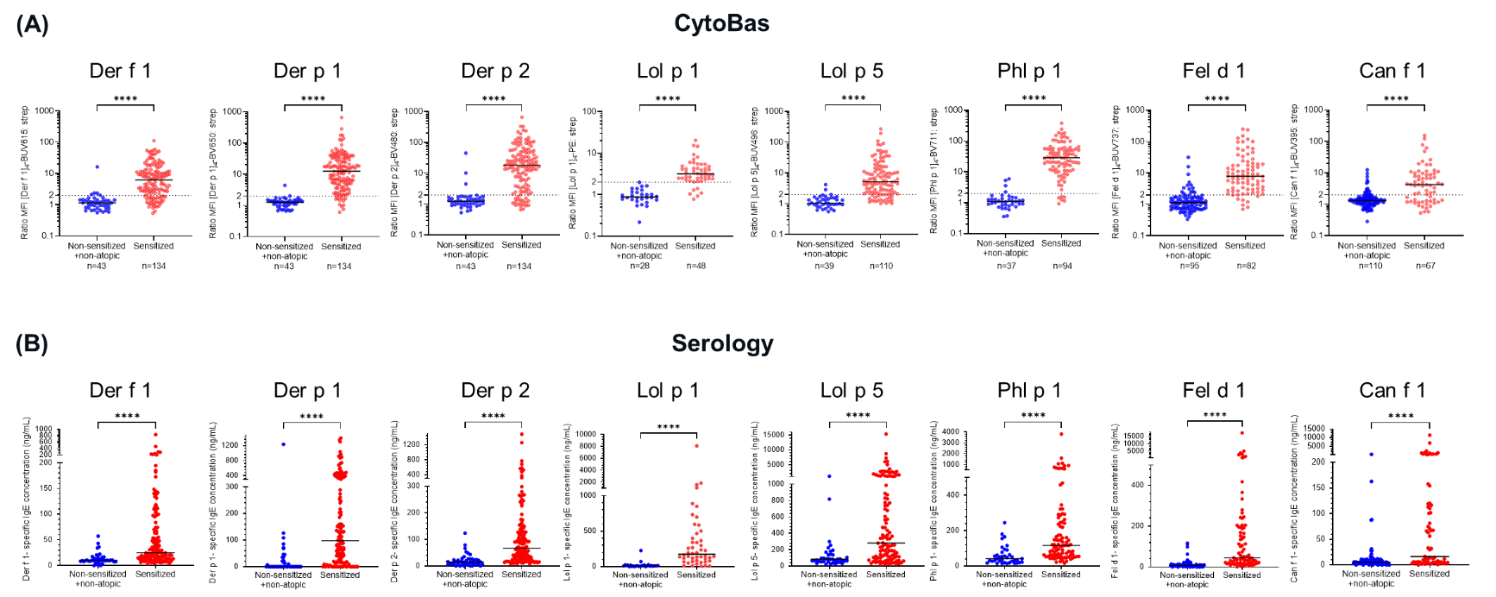


Supplementary Figure 4. Evaluation of staining intensities of AeroDiff CytoBas and specific IgE serology for each aeroallergen component. **(A)** PBMC samples from individuals were evaluated with CytoBas in a single tube. The staining intensity (ratio MFI) on basophils for tetramers [Der f 1]_4_-BUV615, [Der p 1]_4_-BV650, [Der p 2]_4_-BV480, [Lol p 1]_4_-PE, [Lol p 5]_4_-BUV496, [Phl p 1]_4_-BV711, [Fel d 1]_4_-BUV737 and [Can f 1]_4_-BUV395 were compared between sensitized group (in red): allergen-specific ImmunoCAP≥0.35kU/L; and the control group (in blue): allergen-specific ImmunoCAP< 0.35kU/L. Cutoff at the ratio of 2 is indicated by the dotted line. (**B**) Serum specific IgE concentrations to the various recombinant allergens Der f 1, Der p 1, Der p 2, Lol p 1, Lol p 5, Phl p 1, Fel d 1 and Can f 1 were plotted for comparison between the sensitized and control group. Black lines denote median values. Statistics: Mann-Whitney U test; **** p<0.0001.


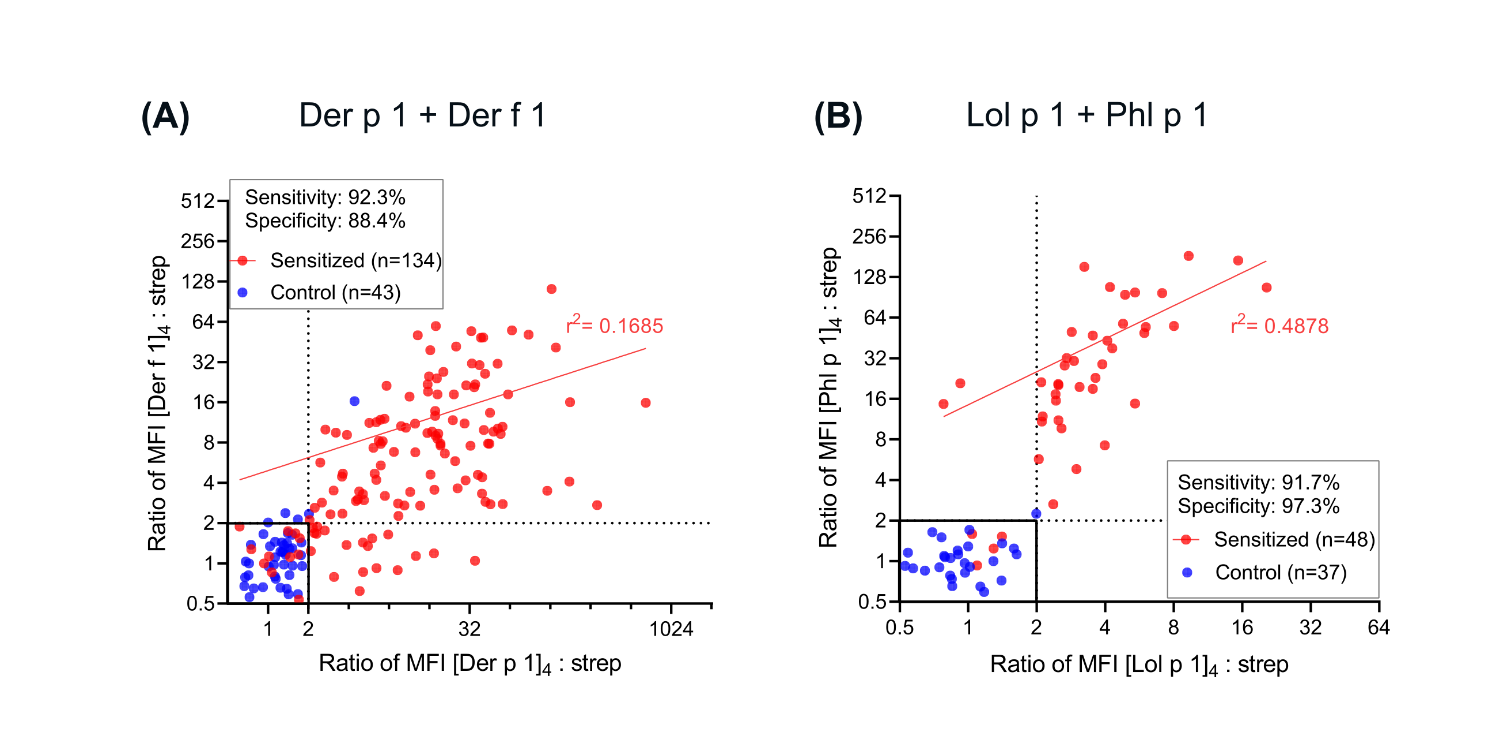
Supplementary Fig 5. Correlation of HDM and GP aeroallergen components with high homology. (**A**) Scatterplot of staining intensity between [Der f 1]_4_-BUV615 and [Der p 1]_4_-BV650 of HDM-sensitized and control samples. The correlation between the tetramer stainings (ratio MFI) in the sensitized group is indicated as the regression line with r-squared (r^2^) of 0.1685. (**B**) Scatterplot of staining intensity between [Lol p 1]_4_-PE and [Phl p 1]_4_-BV711 of RGP-sensitized and control subjects and the correlation between the two allergens only. The non-linear regression line of the sensitized group is illustrated. The cut-off at the ratio MFI of 2 is indicated as dotted lines. Statistics: Log-to log non-linear regression was used to determine the correlation of ratio MFI with r^2^ values indicating the goodness of fit.
